# Lactylation-related gene *AKR1A1* contributes to osteoporosis via metabolic–immune regulation: evidence from multi-omics integration, single-cell transcriptomics, and *in vitro* validation

**DOI:** 10.3389/fimmu.2025.1680305

**Published:** 2025-10-30

**Authors:** Zichen Shao, Qinqin Deng, Ling Cheng, Jianfeng Wu, Weikang Sun, Weidong Liang, Huanan Li

**Affiliations:** ^1^ Jiangxi University of Chinese Medicine, Nanchang, Jiangxi, China; ^2^ Affiliated Hospital of Jiangxi University of Chinese Medicine, Nanchang, Jiangxi, China

**Keywords:** osteoporosis, *AKR1A1*, lysine lactylation, immunometabolism, multi-omics integration, single-cell RNA sequencing, SPP1–CD44 signaling pathway

## Abstract

**Objective:**

This study aimed to systematically identify key differentially expressed genes (DEGs) associated with lysine lactylation in osteoporosis and to explore their potential roles in disease pathogenesis from a dual perspective of metabolic and immune regulation, thereby providing a theoretical basis for targeted therapeutic strategies.

**Methods:**

Five osteoporosis-related gene expression microarray datasets and one single-cell RNA-sequencing dataset were integrated from the GEO database. A lactylation-related gene panel comprising 1,347 genes was used to construct a screening framework. Batch effect correction, differential expression analysis, GO/KEGG enrichment, CIBERSORT-based immune infiltration, and GSEA/GSVA functional annotation were performed. A total of 113 combinations of machine learning models were applied to identify key genes. Single-cell UMAP clustering and CellChat-based intercellular communication analysis were conducted to further characterize the findings. *In vitro* experiments were performed using RAW264.7 macrophages treated with lactate and osteoporotic serum, and gene expression and lactylation levels were validated via qPCR, Western blot, and co-immunoprecipitation (Co-IP).

**Results:**

A total of 37 lactylation-related DEGs were identified, mainly enriched in metabolic and inflammatory pathways. Among them, *AKR1A1* was highlighted as a key feature gene through machine learning models, exhibiting elevated expression and high levels of lactylation, particularly enriched in monocytes and macrophages. CellChat analysis revealed that *AKR1A1* participates in the *SPP1–CD44* signaling pathway, mediating intercellular communication among immune cells. *In vitro* validation confirmed that *AKR1A1* expression and lactylation levels were significantly upregulated under combined lactate and osteoporotic serum treatment, suggesting a synergistic enhancement effect.

**Conclusion:**

*AKR1A1* lactylation plays a pivotal role in the metabolic–immune regulatory axis of osteoporosis, contributing to metabolic reprogramming and immune microenvironment remodeling. Through involvement in the *SPP1–CD44* signaling pathway, it mediates communication between monocytes and macrophages, and may serve as a novel biomarker and therapeutic target for early diagnosis and intervention in osteoporosis.

## 1 Introduction

Osteoporosis (OP) is a systemic skeletal disorder characterized by reduced bone mass and microarchitectural deterioration, leading to increased fracture risk and significant morbidity among the elderly population (1). With the global population aging rapidly, the incidence of OP is steadily rising, posing a substantial public health concern by reducing healthy life expectancy and impairing quality of life. Epidemiological studies estimate that more than 200 million people worldwide are affected by osteoporosis, with approximately one-third of women and one-fifth of men over the age of 50 experiencing at least one osteoporotic fracture (2). Although several pharmacological agents such as bisphosphonates, selective estrogen receptor modulators, and monoclonal antibodies have been approved for the prevention and treatment of OP, their clinical application is often limited by suboptimal efficacy, poor tolerance, and low patient compliance (3, 4). Hence, elucidating the molecular mechanisms underlying OP pathogenesis and identifying novel therapeutic targets remain critical needs.

Recent advances have shifted the paradigm of bone homeostasis research from macroscopic bone mineral density measurements to cellular and molecular mechanisms. Among these, metabolic reprogramming has emerged as a central regulator of bone remodeling processes (5). Lactate, once considered a metabolic waste product of glycolysis, is now recognized as a signaling molecule involved in modulating inflammation, immune responses, and gene transcriptional activity (6, 7). Increasing evidence suggests that lactate plays a pivotal role in bone metabolism through lysine lactylation, a novel post-translational modification (PTM) mechanism (8). Lactylation of histone lysine residues can alter chromatin structure and gene expression, contributing to the dynamic balance between bone formation and resorption. Specifically, lactylation has been shown to promote the transcription of osteogenic genes such as *Runx2* and *JunB*, thereby enhancing osteogenic differentiation of bone marrow mesenchymal stem cells (BMSCs) (9). Simultaneously, lactylation can inhibit osteoclastogenesis and suppress bone resorption. Moreover, lactate modulates the polarization of immune cells, particularly promoting the anti-inflammatory M2 phenotype of macrophages, thereby indirectly influencing bone homeostasis and tissue repair (8). Vascular endothelial cell-derived lactate has also been reported to induce H3K18la histone lactylation in BMSCs, activating osteogenic genes such as *COL1A2* and *COMP*, thereby alleviating osteoporosis progression (10). Collectively, these findings highlight the therapeutic potential of lactylation in bone-related disorders such as osteoporosis and periodontitis. However, the expression patterns and mechanistic roles of lactylation in osteoporosis remain insufficiently characterized, and its involvement in the immunometabolic axis of bone pathology has yet to be systematically explored.

With the continued advancement of omics technologies and data science, integrative multi-omics analysis, machine learning algorithms, and single-cell RNA sequencing (scRNA-seq) have opened new avenues for investigating metabolic bone diseases (11, 12). The integration and batch correction of public transcriptomic datasets enhance the reliability and robustness of DEG identification (13). Machine learning-based feature selection facilitates the efficient discovery of molecular biomarkers with diagnostic or therapeutic relevance (14). Furthermore, single-cell transcriptomic profiling enables the dissection of cellular heterogeneity within the bone marrow microenvironment, and in combination with immune infiltration analysis and intercellular communication networks, allows for a more refined understanding of dynamic regulatory processes *in situ* (15).

In this study, we systematically identified lactylation-related DEGs associated with osteoporosis using publicly available datasets and explored their diagnostic value and functional roles through multi-dimensional bioinformatic analyses. These included immune infiltration profiling, functional enrichment analyses, single-cell expression distribution, and cell–cell communication modeling. Based on these findings, we established an *in vitro* RAW264.7 macrophage model to validate the expression and lactylation levels of key candidate genes (e.g., *AKR1A1*) under different treatment conditions. This integrative strategy—combining multi-omics data mining, machine learning-based gene prioritization, and experimental validation—aims to uncover the mechanistic role of lactylation in the immunometabolic regulation of osteoporosis and to provide a theoretical foundation for future targeted therapeutic interventions.

## 2 Materials and methods

### 2.1 Data acquisition and preprocessing

Microarray datasets related to osteoporosis were retrieved from the Gene Expression Omnibus (GEO) public database (https://www.ncbi.nlm.nih.gov/geo/). A total of five datasets were included in the analysis: GSE7158, GSE56116, GSE56815, GSE7429, and GSE230665. GSE7158 contains 12 low peak bone mass (PBM) samples and 14 high PBM samples (platform: GPL570). GSE56116 includes 10 postmenopausal osteoporosis patients and 3 healthy postmenopausal women (platform: GPL4133). GSE56815 consists of 40 subjects with high hip bone mineral density (BMD) and 40 subjects with low hip BMD (platform: GPL96). GSE7429 includes 10 high BMD and 10 low BMD samples (platform: GPL96). GSE230665 contains 12 postmenopausal osteoporosis patients and 3 healthy postmenopausal controls (platform: GPL10332). Datasets GSE7158, GSE56116, and GSE56815 were merged and designated as the training set, while GSE7429 and GSE230665 were combined to form the validation set. Additionally, single-cell RNA-seq data from human bone marrow-derived mesenchymal stem cells (BMSCs) were obtained from dataset GSE147287, specifically the osteoporosis sample GSM4423510 (platform: GPL24676). A curated list of 1,347 lactylation-related genes was compiled for subsequent analysis.

### 2.2 Reagents and instruments

The reagents and consumables used in this study included: high-glucose DMEM, PBS buffer, trypsin-EDTA, phorbol ester (PMA), Ultrapure RNA Kit, HiFiScript gDNA Removal RT MasterMix, MagicSYBR Mixture, 0.1 mL eight-strip flat-cap tubes, Super Red nucleic acid dye, DNA loading buffer (6X), anhydrous ethanol, chloroform, centrifuge tubes and pipette tips, RIPA lysis buffer, high-efficiency lysis buffer, protease inhibitors, BCA protein assay kit, MOPS-SDS running buffer, FuturePAGE™ pre-cast protein gels, gel preparation kit, dual-color pre-stained protein marker, PVDF membrane, methanol, rapid transfer buffer, nonfat dry milk, QuickBlock™ blocking buffer, primary antibody dilution buffer, secondary antibody dilution buffer, 20×TBST buffer, Meilunbio^®^ Fect ultra-sensitive ECL reagent, AP substrate, Ponceau S staining solution, Coomassie brilliant blue staining solution, and Protein A/G magnetic beads. The main instruments used included: biosafety cabinet, CO_2_ incubator, upright microscope, Gilson P-series pipettes, tissue grinder, ultrasonic cell disruptor, metal bath, shaker, micro-spectrophotometer, PCR gradient thermal cycler, real-time PCR system, gel electrophoresis apparatus, gel documentation system, electrophoresis system, blotting apparatus, PVDF membrane cassette, microplate reader, chemiluminescence imaging system, general imaging system, benchtop high-speed refrigerated centrifuge, low-temperature centrifuge, and water bath.

### 2.3 Differentially expressed genes analysis

To identify DEGs, the training datasets were first subjected to batch effect correction. The limma package in R was employed to perform differential expression analysis between osteoporosis and healthy control samples. Genes with |log_2_ fold change (logFC)| > 0.1375 and adjusted P-value < 0.05 were considered statistically significant DEGs. Heatmaps and volcano plots were generated to visualize the DEGs. For heatmap clustering, the top 50 genes ranked by the absolute value of logFC were selected. The DEGs were then intersected with the 1,347 lactylation-related genes to identify lactylation-associated DEGs.

### 2.4 Gene ontology and Kyoto encyclopedia of genes and genomes enrichment analysis

GO functional enrichment and KEGG pathway analysis of the lactylation-related DEGs were performed using the clusterProfiler package in R. A significance threshold of P < 0.05 and q < 1 was applied to identify enriched biological terms and pathways. The GO analysis covered three main categories: biological process (BP), cellular component (CC), and molecular function (MF).

### 2.5 Machine learning analysis

A comprehensive machine learning framework was employed to systematically evaluate the diagnostic value of lactylation-related DEGs. Gene expression data from the training and validation cohorts were first normalized, followed by the construction of a multi-level machine learning model integrating 113 algorithmic combinations. The foundational algorithms included classical and modern approaches such as Least Absolute Shrinkage and Selection Operator (Lasso), Ridge regression, Elastic Net, Support Vector Machine (SVM), Generalized Linear Model Boosting (glmBoost), Partial Least Squares Generalized Linear Model (plsRglm), Stepwise Generalized Linear Model (Stepglm), Random Forest (RF), Gradient Boosting Machine (GBM), Linear Discriminant Analysis (LDA), Extreme Gradient Boosting (XGBoost), and Naïve Bayes. A two-stage modeling strategy was implemented: in the first stage, different algorithms were used to select feature variables (with a minimum gene threshold of five), and in the second stage, predictive models were constructed based on the selected features. The optimal model was then integrated through multivariate logistic regression, and its performance was assessed by calculating the area under the receiver operating characteristic curve (AUC). This process yielded a combination of lactylation-related DEGs with the best classification performance. To further interpret the model, SHapley Additive exPlanations (SHAP) analysis was applied to quantify each gene’s contribution to prediction outcomes. By calculating SHAP values, genes were ranked according to their importance, thereby elucidating both the positive and negative effects of differential expression on the model’s predictions. This approach provided global and individual-level interpretability of the model. Genes with AUC ≥ 0.7 and ranked among the top five in SHAP importance were identified as key lactylation-related DEGs for downstream analyses. In the training cohort, expression differences of these key genes between osteoporotic and healthy samples were examined. Pearson correlation coefficients were computed to assess relationships among the key lactylation-related DEGs. Finally, a co-expression network of these genes was constructed using the GeneMANIA database, a robust tool for exploring internal functional associations within a gene set.

### 2.6 Gene set enrichment analysis and gene set variation analysis

To explore the biological functions of the key lactylation-related DEGs, both GSEA and GSVA were performed using the KEGG pathway gene sets. For GSEA, enrichment significance was determined using a threshold of P < 0.05. GSVA was employed to assess pathway-level variations across samples, again using KEGG gene sets with the same significance criteria (P < 0.05).

### 2.7 Immune cell infiltration analysis via CIBERSORT

The CIBERSORT algorithm was applied to the training dataset to estimate the proportions of 22 immune cell types in each sample. Samples with P < 0.05 were retained for downstream analysis.

(1) A stacked bar plot was used to illustrate the relative composition of immune cell subsets across samples. (2) Box plots were generated to compare immune cell proportions between osteoporosis and control groups, and intergroup differences were assessed using the Wilcoxon rank-sum test with significance thresholds set at P < 0.001, P < 0.01, and P < 0.05. (3) In the osteoporosis group, Spearman correlation and hierarchical clustering were performed to analyze the interrelationships among immune cell subtypes.

### 2.8 Preprocessing and normalization of single-cell RNA-seq data

Single-cell data preprocessing and normalization were conducted using the Seurat R package. The expression matrix was converted into a Seurat object and filtered to retain cells expressing at least 50 genes and with mitochondrial gene percentages below 5%. Data normalization was performed using the “LogNormalize” method with a scale factor of 10,000. A total of 1,500 highly variable genes were selected based on variance for downstream analyses. FeatureScatter and violin plots were used to validate data quality and expression distribution. Statistical thresholds were set at logFC > 1 and adjusted P-value < 0.05 to ensure both biological relevance and statistical rigor.

### 2.9 Principal component analysis

After standard preprocessing with the ScaleData function, PCA was performed based on the highly variable gene set (identified by VariableFeatures). Twenty principal components (PCs) were extracted. VizDimLoadings plots were used to visualize the contribution of genes to each PC, and DimPlot was employed to visualize sample distributions in reduced dimensions. DimHeatmap was used to display gene expression patterns for the top four PCs. Significance of PCs was assessed using JackStraw analysis with resampling, and the P-value distribution of the top 20 PCs was shown in the JackStrawPlot. Significant PCs were selected for further downstream analysis.

### 2.10 Clustering and visualization of key lactylation-related DEGs

Clustering analysis was performed using the Uniform Manifold Approximation and Projection (UMAP) method. The FindNeighbors and FindClusters functions were applied for cell clustering, with the number of principal components (PCs) set to 20. Dimensionality reduction and visualization were carried out using RunUMAP. Differentially expressed genes (DEGs) in each cluster were identified with the FindAllMarkers function under thresholds of logFC > 1 and P < 0.05. Heatmaps showing the top 10 marker genes per cluster were generated. Cell type annotation was conducted using the SingleR package in conjunction with the HumanPrimaryCellAtlasData reference. Cluster identities were refined using RenameIdents and visualized via UMAP plots. DEGs across annotated cell types were analyzed (logFC > 1, P < 0.05). The expression patterns of key lactylation-related DEGs were further visualized using violin plots (VlnPlot), feature plots (FeaturePlot), and dot plots (DotPlot) to display expression distributions and trends across cell clusters. Pseudotime trajectory analysis was performed using the Monocle 2 package to explore potential differentiation dynamics among distinct cell populations. The normalized expression matrix and cell annotations from the Seurat object were converted into a CellDataSet using the importCDS function. Highly variable genes were selected as ordering genes with setOrderingFilter, and dimensional reduction was carried out with reduceDimension (method = “DDRTree”). Cells were then ordered along pseudotime using orderCells, and branch points were automatically detected to define divergent developmental paths. Cells were visualized by cluster, state, and pseudotime to depict lineage trajectories. The expression patterns of key lactylation-related DEGs (*AKR1A1* and *RRP1B*) were further plotted along the trajectory, revealing their temporal regulation features during cell-fate transitions.

### 2.11 Cell–cell communication analysis

Cell–cell communication analysis was conducted using the CellChat package. A CellChat object was constructed from the single-cell data, incorporating a curated database of human ligand–receptor interactions. Secreted signaling pathways were selected for analysis. After data preprocessing, overexpressed genes and interacting partners were identified for each cell type, and communication probabilities were calculated. Significant signaling interactions were inferred and visualized to represent the number and strength of intercellular communications. For each cell type, communication patterns were independently analyzed, and key ligand–receptor interactions were displayed using bubble plots. Communication networks involving key lactylation-related DEGs were extracted, and their roles within signaling pathways were characterized. Osteoporosis-relevant signaling pathways were selected for detailed visualization using circular layouts, hierarchical layouts, and heatmaps to show interaction intensity and gene expression levels. Contribution analysis was conducted to evaluate the role of ligand–receptor pairs in osteoporosis-associated signaling, and chord diagrams were used to display specific interaction relationships.

### 2.12 Cell induction, grouping, and treatment

The murine macrophage cell line RAW264.7 was selected as the *in vitro* model for functional experiments due to its stable origin, well-characterized phenotype, and widespread use in studies of inflammation and bone metabolism. RAW264.7 cells can be differentiated into M0 macrophages upon induction with phorbol 12-myristate 13-acetate (PMA), making them suitable for stimulation studies under various conditions. A PMA stock solution (100 μM) was diluted to a final concentration of 100 nM with complete culture medium and filtered through a 0.22 μm membrane for use. RAW264.7 cells were digested with trypsin, resuspended at a density of 1×10^6^ cells/mL, and seeded at 1 mL per dish. Cells were incubated overnight at 37 °C to allow adherence. M0 macrophages were generated by treating adherent cells with 100 nM PMA for 48 hours. The cells were then divided into four groups. Ctrl group: conventional culture without treatment. LAC group: treated with lactate (10 mM) for 24 hours. OP group: treated with 10% serum from osteoporosis patients for 24 hours. OP+LAC group: co-treated with 10% osteoporosis patient serum and lactate (10 mM) for 24 hours. Following the treatment period, cells were harvested for reverse transcription quantitative polymerase chain reaction (RT-qPCR), Western blotting, and co-immunoprecipitation (Co-IP) analyses.

### 2.13 RT-qPCR analysis

RT-qPCR was used to quantify the mRNA expression levels of *AKR1A1* in the four experimental groups. Total RNA was extracted and reverse-transcribed into cDNA. Amplification was performed using SYBR Green dye, with *GAPDH* as the internal control. Primer sequences were as follows: *GAPDH*: forward 5′-GCCCAGAACATCATCCCTGCAT-3′, reverse 5′-GCCTGCTTCACCACCTTCTTGA-3′ (product size: 188 bp). *AKR1A1*: forward 5′-AACAGTCGGCAGATTGATGATG-3′, reverse 5′-CCAAGCACGGTCAGAGGAA-3′ (product size: 168 bp). Relative gene expression levels were calculated using the 2^−ΔΔCt method.

### 2.14 Western blot analysis

Western blotting was employed to detect the protein expression levels of *AKR1A1* across the four groups. Total cellular proteins were extracted and quantified using the BCA assay, followed by SDS-PAGE separation and transfer to PVDF membranes. After blocking, membranes were incubated with anti-*AKR1A1* primary antibody and HRP-conjugated secondary antibody. *β-actin* served as the internal control. Bands were visualized using enhanced chemiluminescence (ECL), and grayscale intensity was quantified using ImageJ software to determine the relative expression levels of *AKR1A1*.

### 2.15 Co-immunoprecipitation assay

Co-IP was performed to assess the lactylation level of *AKR1A1* in the four treatment groups. Cells were lysed in IP lysis buffer containing protease inhibitors, followed by grinding, ultrasonication, and centrifugation to obtain total protein. Pre-clearing was conducted with magnetic beads to remove non-specific proteins. Subsequently, anti-*AKR1A1* antibody or IgG control was added to the lysate and incubated at 4°C to form immune complexes, which were then incubated with magnetic beads. After washing, beads were resuspended in reducing loading buffer and boiled at 100 °C to elute the immunoprecipitated (IP) samples, with the remaining input used as control. For Western blot detection, samples were separated via SDS-PAGE and transferred to membranes. Membranes were blocked and incubated with anti-*AKR1A1* or anti-pan-Kla (pan-lysine lactylation) antibodies, followed by HRP-conjugated secondary antibodies. Signal detection was performed using ECL, and band intensities were quantified with ImageJ to assess *AKR1A1* lactylation levels.

## 3 Results

### 3.1 Identification of lactylation-related DEGs in osteoporosis

**Figure 1:**
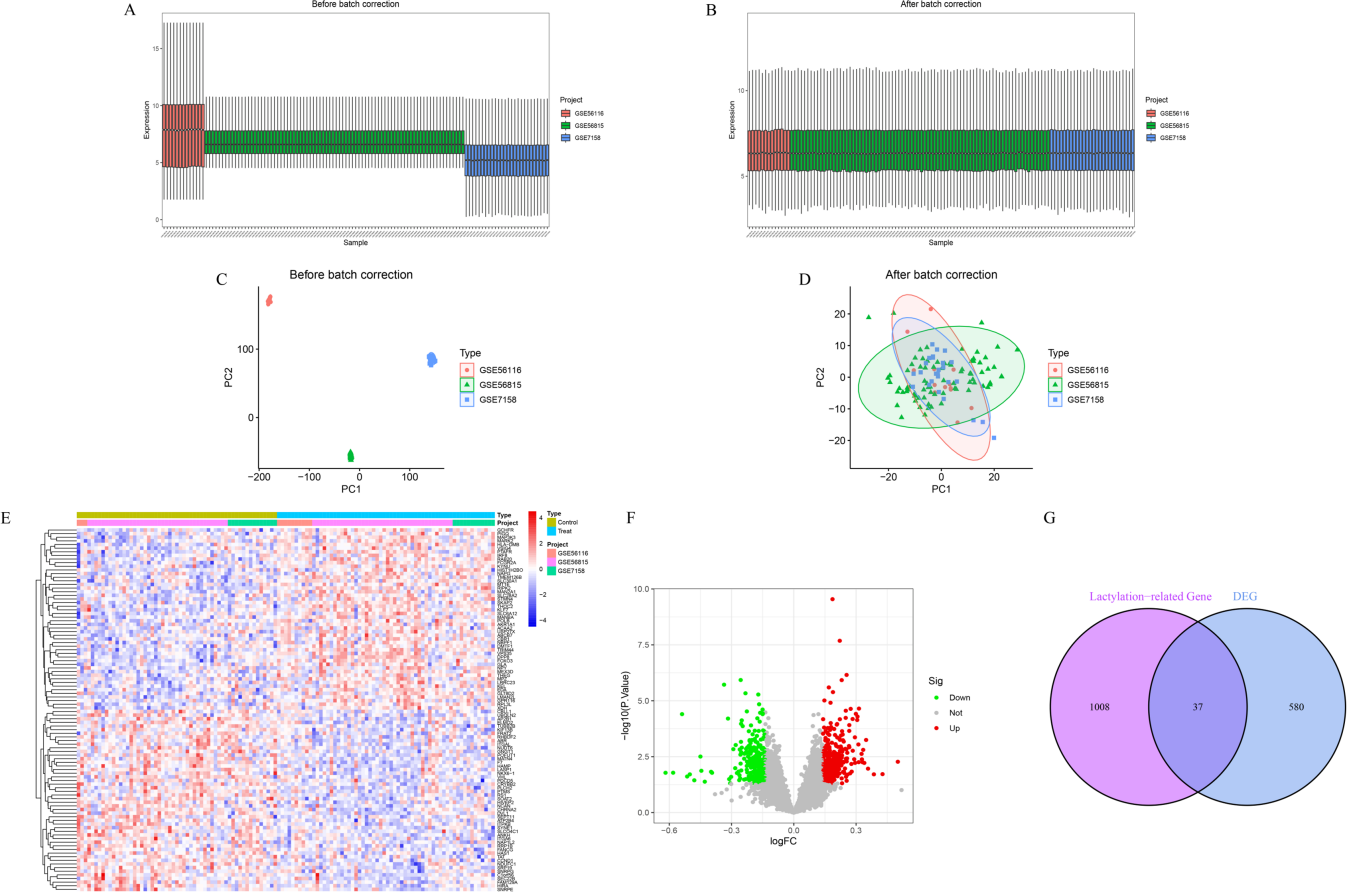
Batch effect correction and identification of DEGs. **(A, B)** Boxplots of gene expression before **(A)** and after **(B)** batch correction. **(C, D)** PCA plots before **(C)** and after **(D)** batch correction. **(E)** Heatmap of top 50 DEGs ranked by absolute logFC. **(F)** Volcano plot showing the distribution of all DEGs. **(G)** Venn diagram showing the overlap between DEGs and lactylation-related genes.

Prior to batch correction, expression boxplots (**Figure 1A**) and PCA scatter plots (**Figure 1C**) revealed pronounced sample clustering and expression heterogeneity across datasets, indicating significant batch effects. After correction, the data distribution was markedly improved (**Figures 1B, D**), and PCA confirmed that inter-sample variance was minimized, indicating successful removal of batch effects. A total of 617 differentially expressed genes (DEGs) were identified between osteoporosis and healthy control samples, including 330 upregulated and 287 downregulated genes. Heatmap and volcano plots illustrating these DEGs are shown in **Figures 1E, F**. By intersecting the 617 DEGs with 1,347 lactylation-related genes, we identified 37 lactylation-related DEGs (**Figure 1G**).

### 3.2 GO functional and KEGG pathway enrichment analysis

**Figure 2:**
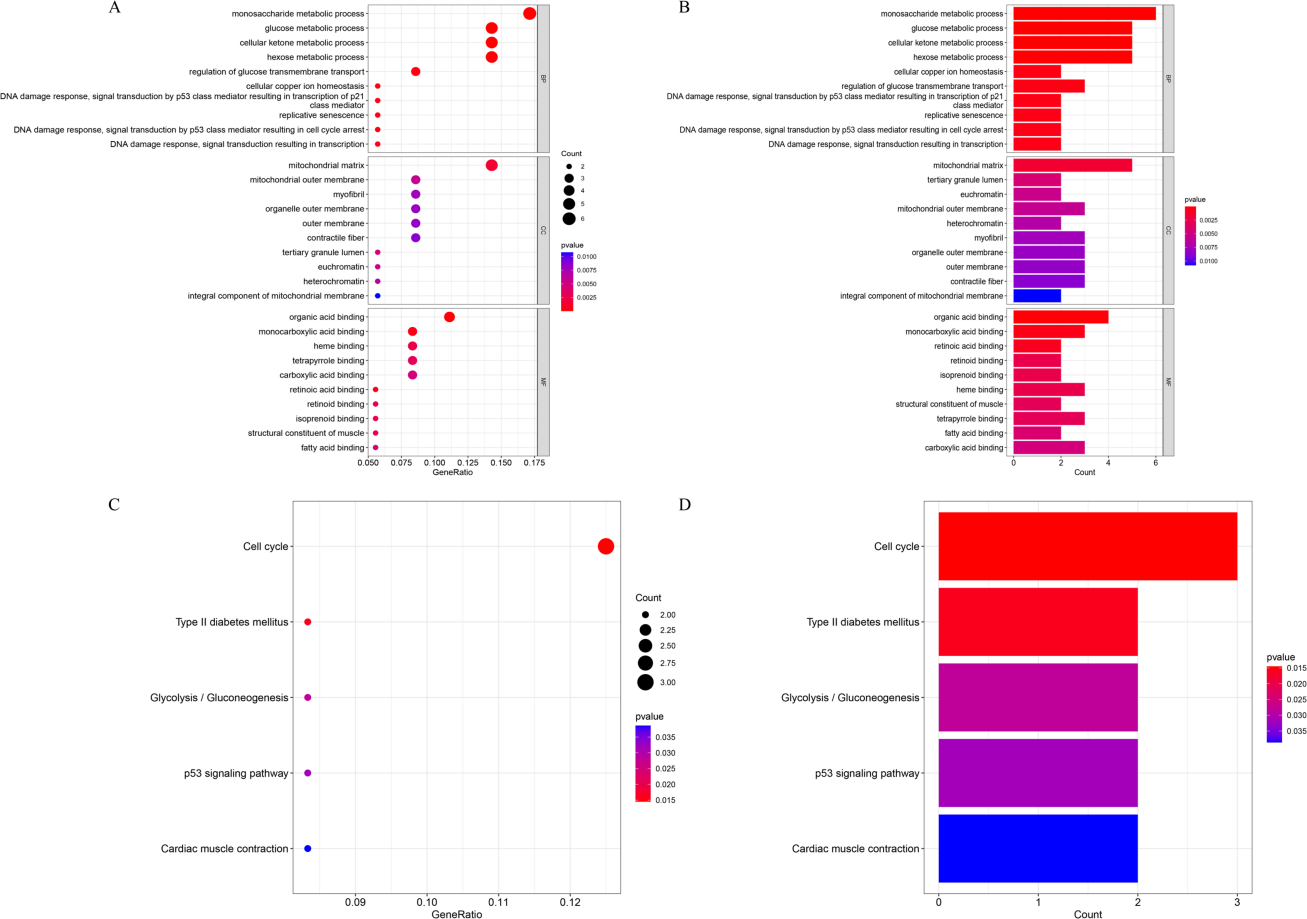
Enrichment analysis of lactylation-related DEGs. **(A, B)** GO enrichment results presented as a bubble plot **(A)** and bar chart **(B)**. **(C, D)** KEGG pathway enrichment results shown as a bubble plot **(C)** and bar chart **(D)**.

GO enrichment analysis of the 37 lactylation-related DEGs revealed significant involvement in glucose metabolic processes, including monosaccharide metabolism, glucose metabolism, cellular ketone metabolism, hexose metabolism, and regulation of transmembrane glucose transport (**Figures 2A, B**). KEGG pathway enrichment indicated that these genes were associated with cell cycle regulation, type II diabetes mellitus, glycolysis/gluconeogenesis, the p53 signaling pathway, and cardiac muscle contraction (**Figures 2C, D**).

### 3.3 Identification of key lactylation-related DEGs via 113 machine learning model combinations

**Figure 3:**
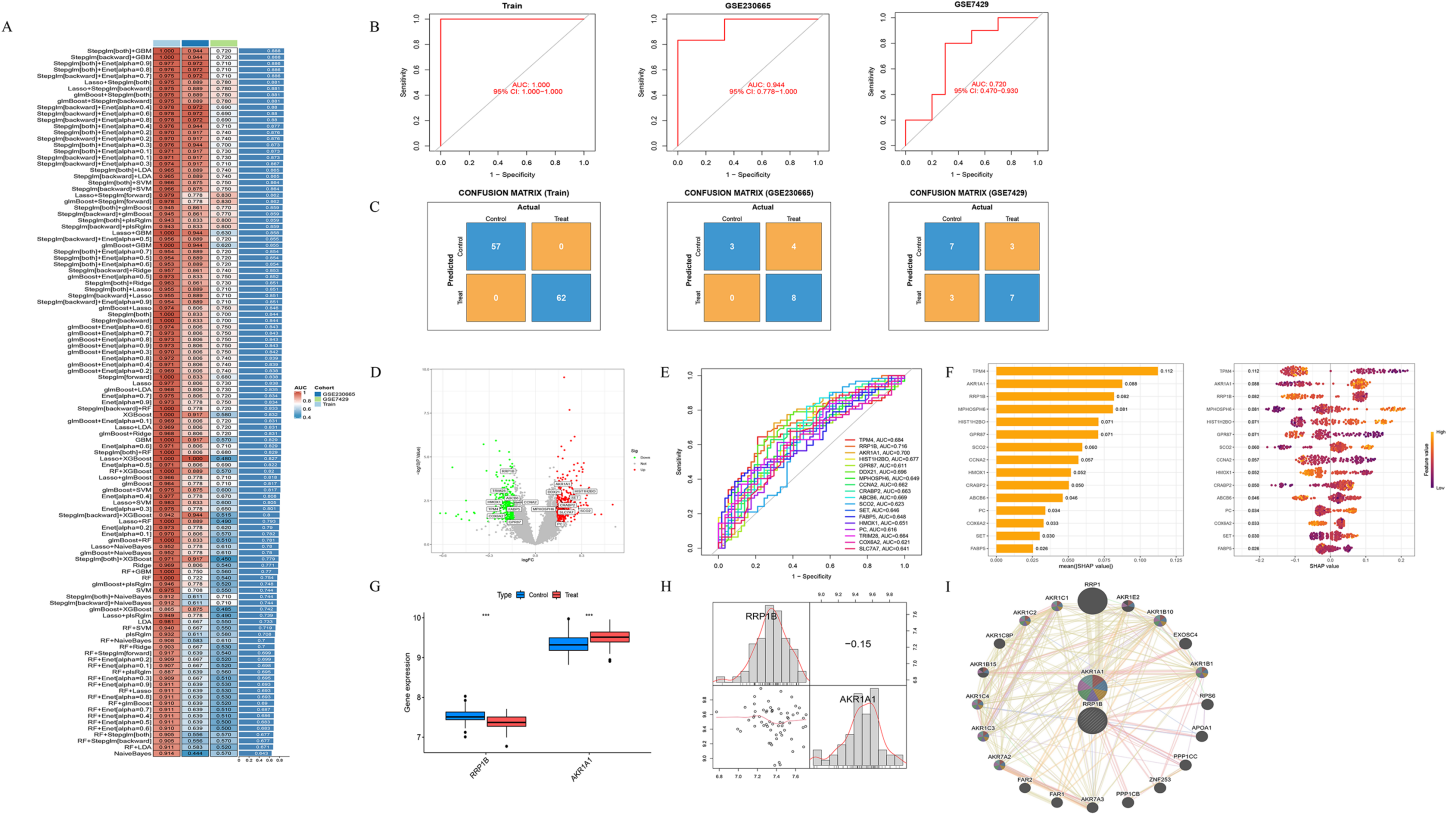
Screening of key lactylation-related DEGs. **(A)** Distribution of AUC values and performance heatmap of 113 models constructed using multiple machine learning algorithms, identifying Stepglm[both]+GBM and Stepglm[backward]+GBM as the optimal models. **(B)** Receiver operating characteristic (ROC) curves and corresponding AUC values of the optimal models in the training cohort, GSE230665 validation cohort, and GSE7429 validation cohort. **(C)** Confusion matrices of the optimal models across the training cohort and two validation cohorts. **(D)** Volcano plot of 18 lactylation-related DEGs identified by the optimal model. **(E)** ROC curves of the 18 lactylation-related DEGs. **(F)** SHAP value analysis of key feature genes within the GBM model. **(G)** Boxplots showing the expression levels of *RRP1B* and *AKR1A1* in osteoporotic and healthy samples. **(H)** Scatterplot matrix illustrating the relationship between *RRP1B* and *AKR1A1*, including histogram distributions, kernel density curves, and the Pearson correlation coefficient (r = −0.15). **(I)** Co-expression network of *RRP1B* and *AKR1A1*, displaying their associated genes and functional annotations.

A multi-level machine learning framework encompassing 113 algorithmic combinations was established to identify key lactylation-related DEGs, with model performance evaluated using the area under the receiver operating characteristic curve (AUC). The results demonstrated that the Stepglm[both]+GBM and Stepglm[backward]+GBM models achieved optimal performance compared with other algorithmic combinations. Both models exhibited stable and robust performance across the training cohort (AUC = 1.000), the independent validation cohort GSE230665 (AUC = 0.944), and the validation cohort GSE7429 (AUC = 0.720), yielding an average AUC of 0.888 (**Figures 3A–C**). Based on these optimal models, a set of 18 lactylation-related DEGs with the highest classification performance was identified, including *TPM4*, *RRP1B*, *AKR1A1*, *HIST1H2BO*, *GPR87*, *DDX21*, *MPHOSPH6*, *CCNA2*, *CRABP2*, *ABCB6*, *SCO2*, *SET*, *FABP5*, *HMOX1*, *PC*, *TRIM28*, *COX6A2*, and *SLC7A7* (**Figure 3D**). Among these, *RRP1B* and *AKR1A1* were selected as key lactylation-related DEGs for further analysis, as they exhibited both AUC ≥ 0.7 and top-five SHAP importance rankings (**Figures 3E, F**). In the training dataset, *RRP1B* was significantly downregulated in osteoporotic samples compared with healthy controls (P < 0.001), while *AKR1A1* was markedly upregulated in osteoporotic samples (P < 0.001) (**Figure 3G**). Correlation analysis between the two genes revealed a weak negative association (r = −0.15, P > 0.05) (**Figure 3H**). Using the GeneMANIA database, we further explored the co-expression and functional interaction network of *RRP1B* and *AKR1A1*. The two genes were involved in a complex protein–protein interaction (PPI) network, in which physical interactions accounted for 77.64%, co-expression for 8.01%, predicted associations for 5.37%, co-localization for 3.63%, genetic interactions for 2.87%, pathway relationships for 1.88%, and shared protein domains for 0.60% (**Figure 3I**).

### 3.4 GSEA and GSVA analyses of key genes

**Figure 4:**
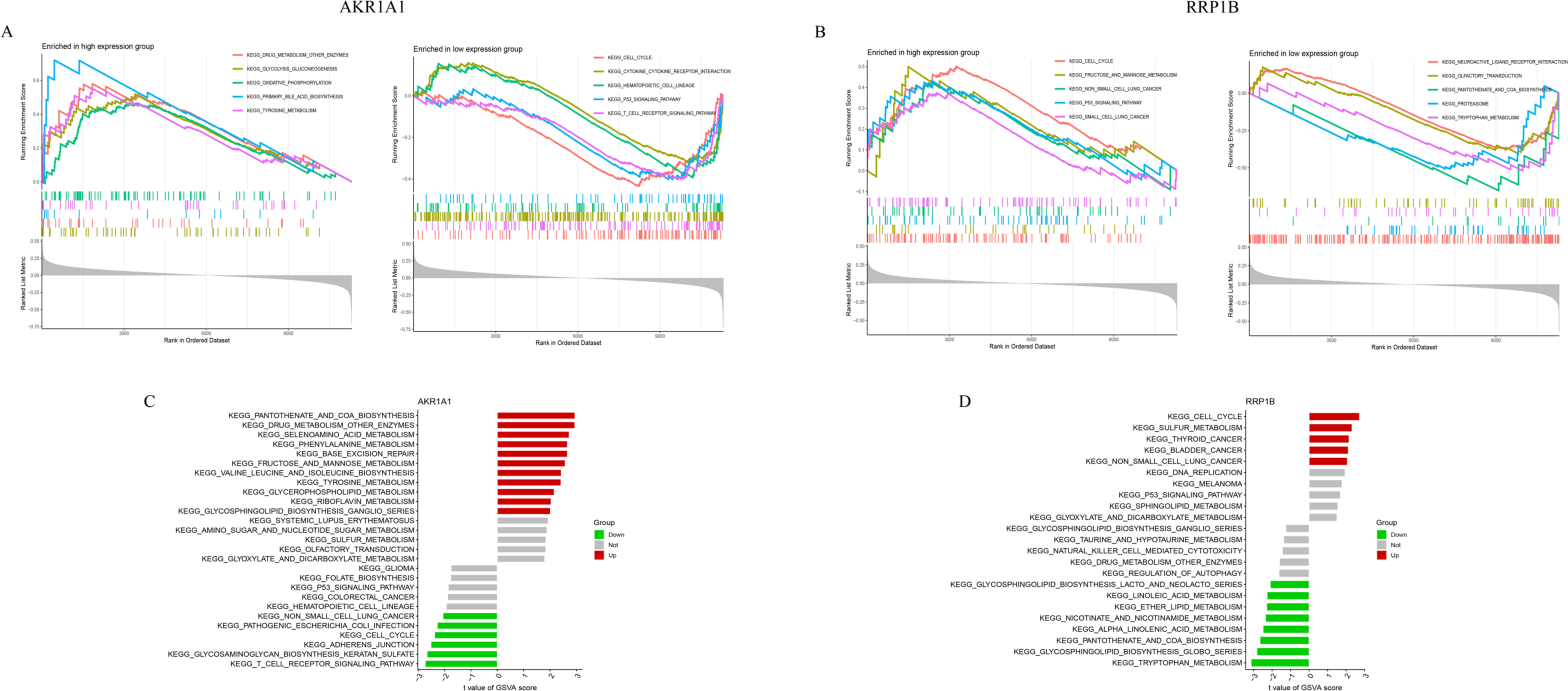
Functional enrichment of *AKR1A1* and *RRP1B* by GSEA and GSVA. **(A)** GSEA results comparing high- and low-expression groups of *AKR1A1*. **(B)** GSEA results for *RRP1B*. **(C)** GSVA enrichment pathways for *AKR1A1*. **(D)** GSVA enrichment pathways for *RRP1B*.

GSEA was performed to explore the pathway enrichment characteristics of high- and low-expression subgroups of *AKR1A1* and *RRP1B* (**Figures 4A, B**). In the *AKR1A1* low-expression group, pathways such as cell cycle, cytokine–cytokine receptor interaction, hematopoietic cell lineage, p53 signaling, and T cell receptor signaling were enriched. Conversely, the high-expression group was enriched in pathways related to drug metabolism, glycolysis/gluconeogenesis, oxidative phosphorylation, primary bile acid biosynthesis, and tyrosine metabolism (**Figure 4A**). For *RRP1B*, the low-expression group was enriched in neuroactive ligand–receptor interaction, olfactory transduction, ubiquitin-mediated proteolysis, pantothenate and CoA biosynthesis, and tryptophan metabolism. The high-expression group was enriched in cell cycle, fructose and mannose metabolism, non-small cell lung cancer, p53 signaling pathway, and small cell lung cancer (**Figure 4B**). GSVA was further applied to systematically assess functional pathway enrichment in different expression groups of *AKR1A1* and *RRP1B* (**Figures 4C, D**). The *AKR1A1* high-expression group was enriched in various metabolic pathways including pantothenate and CoA biosynthesis, drug metabolism (other enzymes), selenoamino acid metabolism, phenylalanine metabolism, nucleotide sugar metabolism, and fructose/mannose metabolism. The low-expression group was enriched in cancer-related pathways such as non-small cell lung cancer, colorectal cancer, cell cycle, and T cell receptor signaling (**Figure 4C**). In the *RRP1B* high-expression group, enrichment was observed in cell cycle, sulfur metabolism, thyroid cancer, bladder cancer, and non-small cell lung cancer pathways. The low-expression group was enriched in lipid and amino acid metabolic pathways including sphingolipid biosynthesis, linoleic acid metabolism, ether lipid metabolism, nicotinate metabolism, pantothenate and CoA biosynthesis, and tryptophan metabolism (**Figure 4D**).

### 3.5 Immune infiltration analysis via CIBERSORT

**Figure 5:**
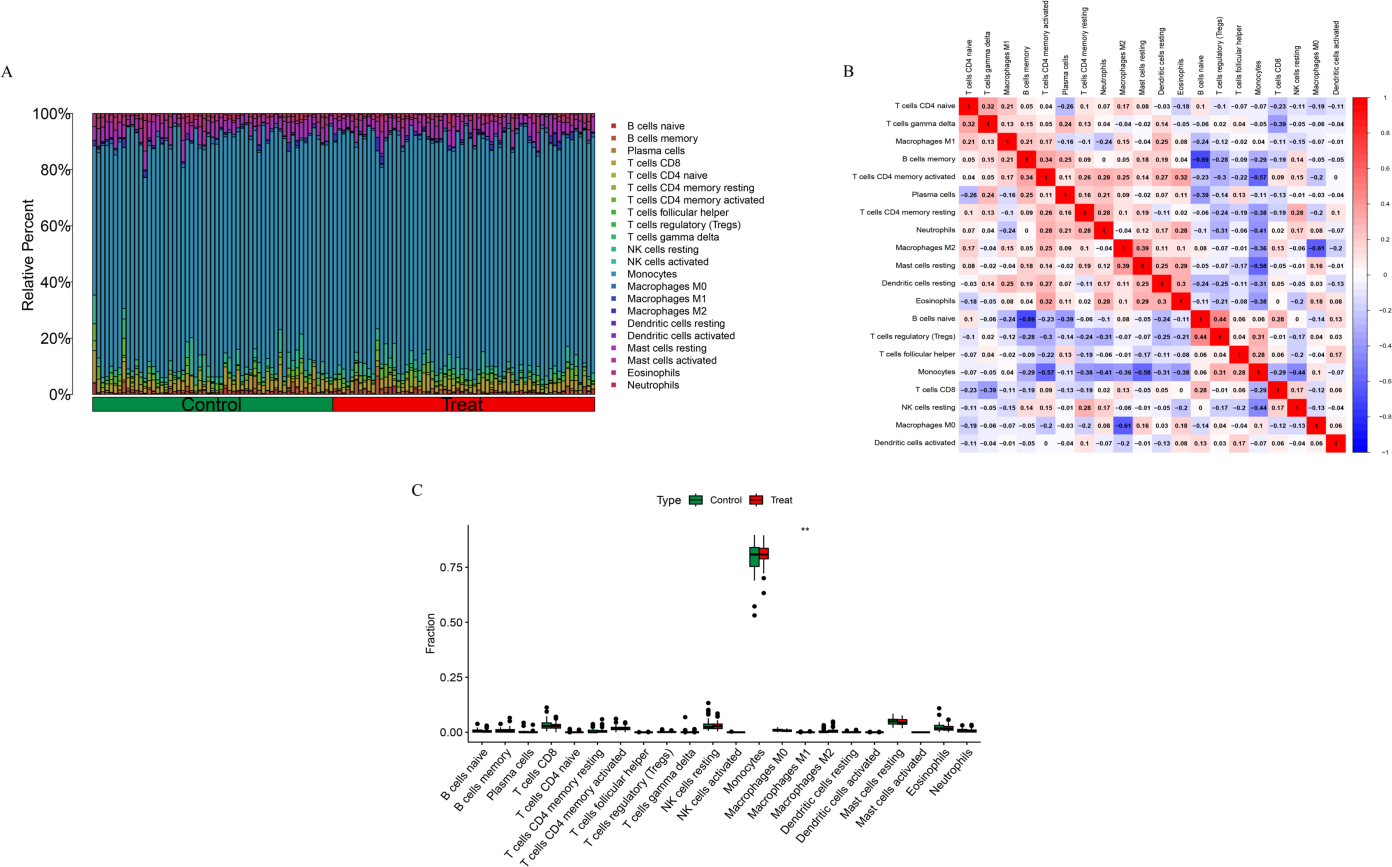
Immune cell infiltration analysis results. **(A)** Stacked bar chart showing the relative proportions of 22 immune cell subtypes in the Control and Treat groups. **(B)** Spearman correlation heatmap among immune cell subsets. **(C)** Boxplots comparing immune cell proportions between the Control and Treat groups. **p < 0.01.

The CIBERSORT algorithm was used to evaluate the immune cell infiltration landscape in the training dataset. We compared the composition of immune cells between the healthy control (Control) and osteoporosis (Treat) groups (**Figure 5A**). Monocytes were the most abundant immune cell type across all samples, suggesting their predominant role in the local immune milieu. Macrophage subsets (M0, M1, and M2) were also widely distributed in both groups. A Spearman correlation heatmap was constructed to visualize interrelationships among immune cell subsets (**Figure 5B**). The results indicated both positive and negative correlations between various cell types. Notably, T cell subsets (e.g., CD4 naive T cells, CD4 memory resting T cells) showed a negative correlation with macrophage subsets (M0, M1, and M2), suggesting potential reciprocal regulation between T cell infiltration and macrophage dynamics. Monocytes were positively correlated with all three macrophage subsets, implying that monocytes may serve as a source for macrophage infiltration and polarization. Boxplot analysis (**Figure 5C**) revealed that the proportion of M1 macrophages was significantly elevated in the osteoporosis group (P < 0.01), while no statistically significant differences were observed for other immune cell types (p > 0.05).

### 3.6 Clustering and visualization of key lactylation-related DEGs

**Figure 6:**
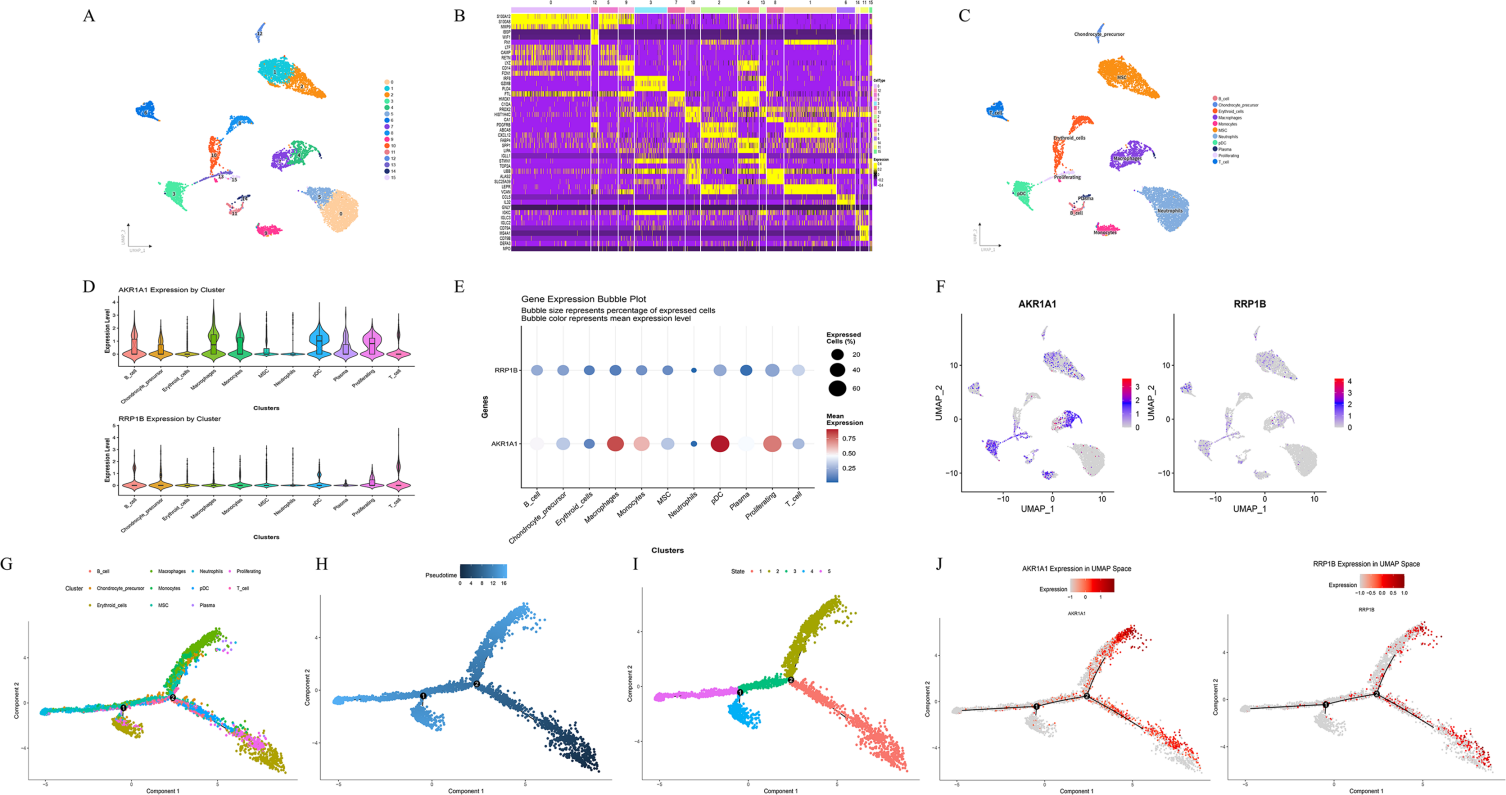
Clustering, annotation, and pseudotime analysis of key lactylation-related DEGs. **(A)** UMAP visualization of 16 identified cell clusters using the FindClusters function. **(B)** Heatmap showing the top 10 highly expressed marker genes per cluster. **(C)** Cell type annotation results displaying major cell populations including B_cell, Chondrocyte_precursor, Erythroid_cells, Macrophages, Monocytes, MSC, Neutrophils, pDC, Plasma, Proliferating, and T_cell. **(D–F)** Expression distribution of key lactylation-related DEGs (*AKR1A1* and *RRP1B*) visualized by violin plots, dot plots, and feature plots. **(G–I)** Pseudotime trajectory analysis showing differentiation dynamics among distinct cell populations, with cells ordered by cluster, pseudotime, and state. **(J)** Expression trends of *AKR1A1* and *RRP1B* along the pseudotime trajectory.

Dimensionality reduction and clustering were performed using the Uniform Manifold Approximation and Projection (UMAP) algorithm. A total of 16 cell clusters were identified with the FindClusters function, and the UMAP plot illustrated their spatial distribution, with each color representing a distinct cell population (**Figure 6A**). Differentially expressed marker genes for each cluster were determined using the FindAllMarkers function, and the top 10 highly expressed marker genes per cluster were visualized in a heatmap (**Figure 6B**). Cell-type annotation revealed that the sample mainly contained B cells, chondrocyte precursors, erythroid cells, macrophages, monocytes, mesenchymal stem cells (MSC), neutrophils, plasmacytoid dendritic cells (pDC), plasma cells, proliferating cells, and T cells (**Figure 6C**). Visualization of key lactylation-related genes showed that *AKR1A1* was markedly upregulated in macrophages, monocytes, pDCs, and proliferating cells, whereas *RRP1B* was predominantly upregulated in T cells (**Figures 6D–F**). Pseudotime trajectory analysis demonstrated clear differentiation trends along the developmental continuum among distinct cell clusters (**Figures 6G–I**). The overall trajectory exhibited a branched tree-like structure, suggesting multiple potential differentiation routes. Cells progressively transitioned from early to mature states, with major trajectories indicating differentiation from MSCs and chondrocyte precursors toward immune-related subpopulations such as macrophages, monocytes, and neutrophils. In the state-based visualization, cells were divided into five states (State 1–5); early-stage cells were mainly distributed in MSCs and erythroid cells, whereas late-stage cells were enriched in macrophages and monocytes, indicating a distinct temporal pattern of lineage progression. Further analysis revealed that *AKR1A1* expression markedly increased during the late pseudotime stages (mainly corresponding to macrophage and monocyte phases), whereas *RRP1B* exhibited a relatively stable expression pattern without pronounced time dependence (**Figure 6J**).

### 3.7 Results of cell–cell communication analysis

**Figure 7:**
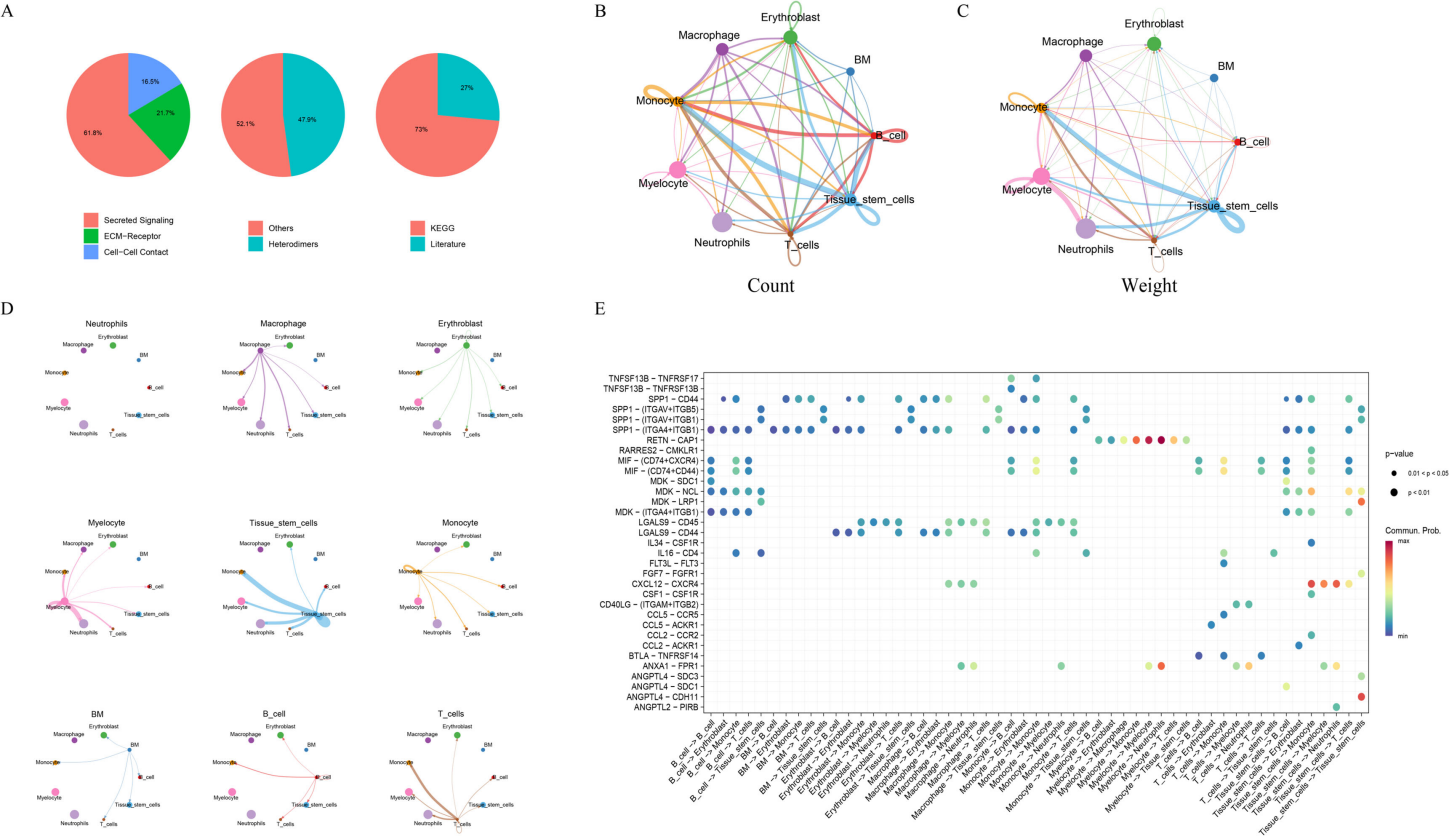
Cell–cell communication network inferred from single-cell RNA-seq data. **(A)** Proportional distribution of signaling modalities, including secreted signaling, ECM–receptor interaction, and cell–cell contact. **(B, C)** Cell–cell interaction counts **(B)** and strengths **(C)** among different cell subsets. **(D)** Network of ligand–receptor pairs between cell types showing interaction directionality. **(E)** Bubble plot of key ligand–receptor pairs. The x-axis indicates sender–receiver pairs; the y-axis indicates ligand–receptor pairs; bubble color represents communication probability; and bubble size denotes statistical significance (P-value).

**Figure 8:**
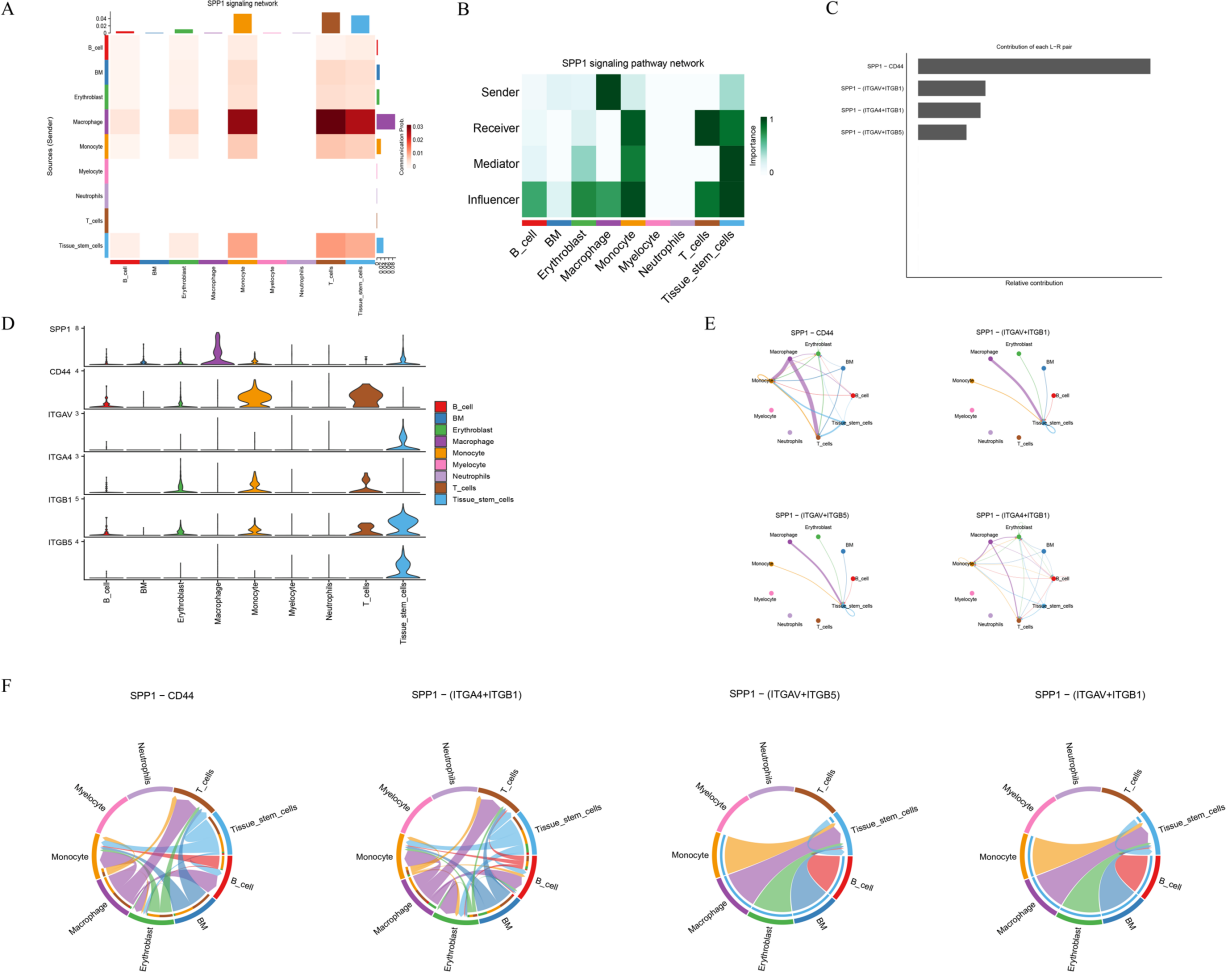
*SPP1* signaling pathway network analysis. **(A)** Heatmap of cell–cell communication strength for the *SPP1* pathway. **(B)** Functional roles of cell types in *SPP1* signaling (Sender, Receiver, Mediator, Influencer). **(C)** Contribution scores of major ligand–receptor pairs within the *SPP1* network. **(D)** Violin plots showing expression levels of *SPP1*, *CD44*, *ITGAV*, *ITGA4*, *ITGB1*, and *ITGB5* across cell types. **(E)** Network diagram of cell interactions via key *SPP1*-related ligand–receptor pairs. **(F)** Chord diagram illustrating *SPP1* signaling interactions.

Cell–cell communication analysis was performed using the CellChat package to construct interaction networks based on ligand–receptor pairs. The results revealed that intercellular communication occurred primarily through three major modalities: secreted signaling (61.8%), extracellular matrix (ECM)–receptor interactions (21.7%), and cell–cell contact (16.5%) (**Figure 7A**). Among the cell populations, Monocytes and Macrophages exhibited particularly active interactions, reflecting robust communication activity. Quantitative evaluation of interaction count (**Figure 7B**) and communication strength (**Figure 7C**) indicated that the interaction frequency and intensity between Monocytes and Macrophages were remarkably elevated, suggesting a key role for these cell types in modulating the immune microenvironment. A ligand–receptor network illustrating directional relationships among different cell types was constructed (**Figure 7D**), revealing major signaling pairs across cell subsets. The bubble plot displayed the critical ligand–receptor interactions along with their corresponding communication probabilities and P-values (**Figure 7E**). Notably, ligand–receptor pairs from Macrophages to Monocytes included *CXCL12–CXCR4*, *LGALS9–CD44*, *LGALS9–CD45*, *SPP1–(ITGA4+ITGB1)*, and *SPP1–CD44*. All detected signaling pathways were identified, including *SPP1*, *RESISTIN*, *CXCL*, *MK*, *ANNEXIN*, *MIF*, *ANGPTL*, *GALECTIN*, *IL16*, *FGF*, *BAFF*, *CD40*, *CCL*, *CHEMERIN*, *CSF*, *BTLA*, and *FLT3*. Given that *SPP1* plays a direct role in bone remodeling by modulating osteoblast and osteoclast activity, it was selected for further analysis. The major cell types participating in the *SPP1* signaling network included B cells, BM cells, Erythroblasts, Macrophages, Monocytes, Myelocytes, Neutrophils, T cells, and Tissue stem cells. A heatmap revealed strong signaling between Macrophages and Monocytes (**Figure 8A**). Role analysis showed that Macrophages functioned as Senders and Influencers, while Monocytes acted as Mediators, Influencers, and Receivers in the *SPP1* pathway (**Figure 8B**). Contribution analysis of ligand–receptor interactions highlighted *SPP1–CD44* as a key signaling axis (**Figure 8C**). Violin plots showed that *SPP1* was highly expressed in Macrophages, while *CD44* was enriched in Monocytes (**Figure 8D**). The chord diagram and network plot further demonstrated frequent and directional communication, particularly between Macrophages and Monocytes (**Figures 8E, F**), indicating that the *SPP1–CD44* axis may serve as a critical bridge in their interaction.

### 3.8 Changes in RAW264.7 cell morphology, *AKR1A1* expression, and lactylation levels under lactate intervention

**Figure 9:**
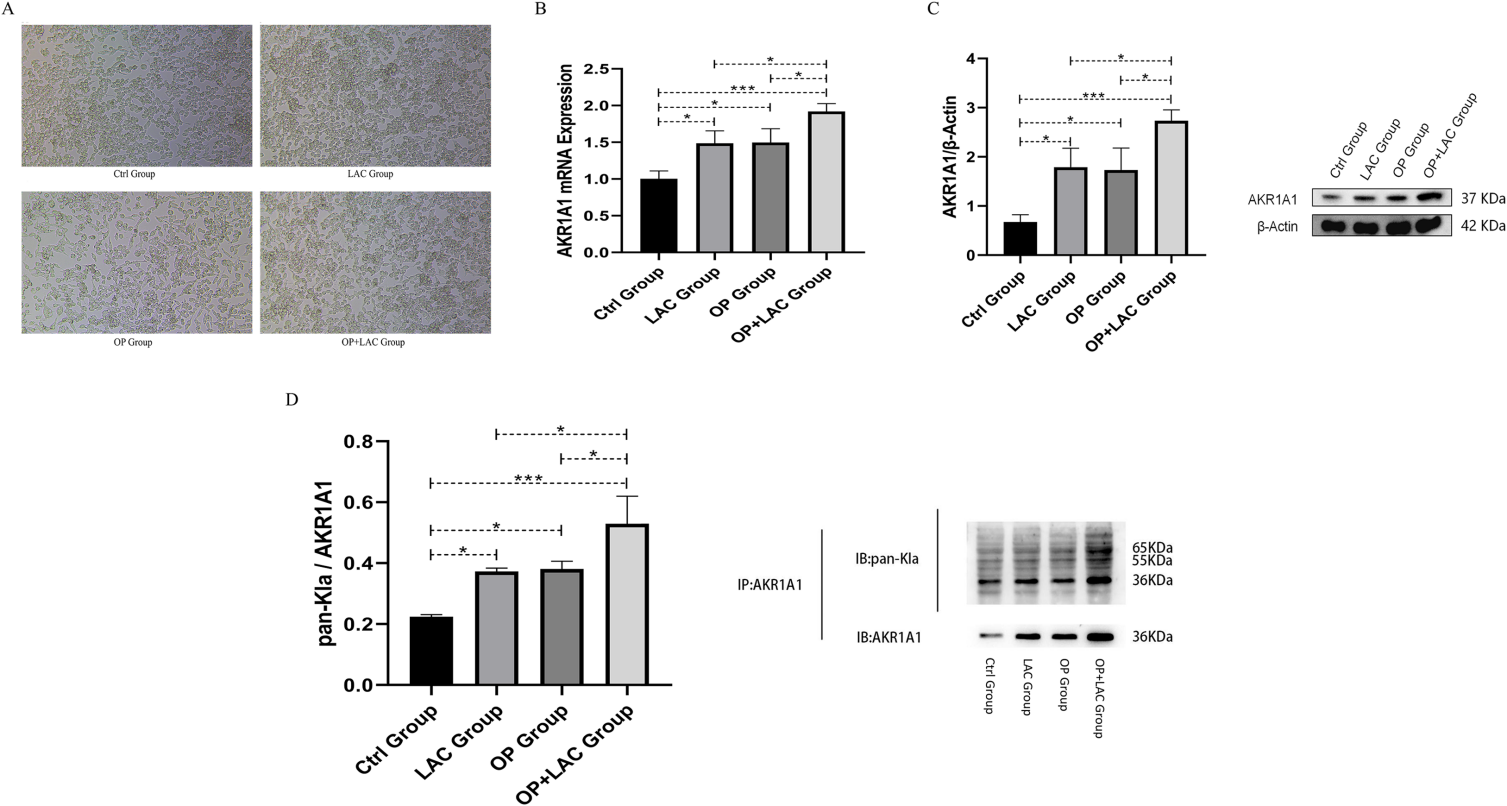
orphological changes, *AKR1A1* expression, and lactylation levels in RAW264.7 cells under lactate intervention. **(A)** Morphological alterations of RAW264.7 cells under different treatment conditions (optical microscopy, ×200). Ctrl group: normal culture; LAC group: treated with lactate (10 mM) for 24 h; OP group: treated with serum from osteoporotic patients (10%) for 24 h; OP+LAC group: co-treatment with osteoporotic patient serum (10%) and lactate (10 mM) for 24 h. **(B)** mRNA expression levels of *AKR1A1* in the four groups of RAW264.7 cells. **(C)** Protein expression levels of *AKR1A1* in the four groups of RAW264.7 cells, with *β-actin* used as an internal reference. **(D)** Lactylation levels of *AKR1A1* in the four groups. *AKR1A1* was used as the immunoprecipitation antibody (IP: *AKR1A1*), and pan-Kla antibody was used to detect lactylation modification (IB: pan-Kla). The lower panel shows the internal control for *AKR1A1* expression (IB: *AKR1A1*). *p < 0.05, **p < 0.01, ***p < 0.001.

After 48 hours of PMA (100 nM) induction, RAW264.7 cells exhibited adherent growth with a uniform morphology, indicating successful differentiation into M0-type macrophages. Distinct morphological differences were observed under different treatment conditions. In the control group (Ctrl), cells were densely arranged with regular morphology. The lactate-treated group (LAC) showed a comparable density to the control, with clear boundaries and slightly shrunken cell bodies. In contrast, the osteoporosis serum group (OP) exhibited reduced adhesion, sparse distribution, and irregular shapes, with some cells showing shrinkage or detachment. Notably, the OP+LAC group demonstrated markedly improved morphology compared with the OP group, characterized by enhanced adhesion and tighter arrangement (**Figure 9A**). These findings suggest that lactate exerts a protective and regulatory effect on RAW264.7 cell morphology under osteoporosis-related stress.

RT-qPCR analysis revealed significant differences in *AKR1A1* mRNA expression among the four groups (**Figure 9B**). Compared with the Ctrl group, both the LAC and OP groups exhibited significantly increased *AKR1A1* mRNA levels (P < 0.05), with the OP+LAC group showing the most pronounced elevation (P < 0.001). Further comparison indicated that *AKR1A1* expression in the OP+LAC group was significantly higher than in either the LAC or OP group (both P < 0.05), while no statistical difference was observed between the LAC and OP groups (P > 0.05). These results indicate that both lactate and osteoporosis serum upregulate *AKR1A1* transcription, and their combined treatment produces a synergistic enhancement effect.

Western blot analysis showed results consistent with the mRNA expression pattern (**Figure 9C**). Compared with the Ctrl group, *AKR1A1* protein expression was significantly increased in both the LAC and OP groups (P < 0.05), with the OP+LAC group showing a further marked increase (P < 0.001). Protein expression in the OP+LAC group was significantly higher than in either the LAC or OP group (both P < 0.05), while no significant difference was observed between the latter two (P > 0.05). These findings demonstrate that both lactate and osteoporosis serum promote *AKR1A1* protein expression, with a clear synergistic effect when combined.

To further assess lactylation modification of *AKR1A1*, co-immunoprecipitation (Co-IP) was performed to detect the relative expression of pan-Kla/*AKR1A1* (**Figure 9D**). Compared with the Ctrl group, lactylation levels were significantly elevated in the LAC and OP groups (P < 0.05) and were most pronounced in the OP+LAC group (P < 0.001). The lactylation level in the OP+LAC group was significantly higher than in either the LAC or OP group (both P < 0.05), whereas no significant difference was found between the LAC and OP groups (P > 0.05). These results suggest that under osteoporotic conditions, lactate intervention further enhances *AKR1A1* lactylation modification, with the combined effects of lactate and osteoporotic serum exhibiting a pronounced synergistic upregulation.

## 4 Discussion

Osteoporosis is a prototypical age-related chronic bone metabolic disorder, traditionally characterized by an imbalance between bone formation and resorption. In recent years, the emerging concept of “immunometabolism” has garnered increasing attention, highlighting the pivotal role of immune cell metabolic reprogramming in modulating the inflammatory microenvironment of bone tissue (16, 17). Lactate, a terminal product of glycolysis, not only functions as a metabolic intermediate but also acts as an epigenetic regulator via protein lactylation (Kla), thereby influencing chromatin remodeling and gene transcription (18). Zhang et al. were the first to identify lactylation modifications on histone lysine residues, revealing its role in gene activation (19). Subsequent studies demonstrated that Kla exerts broad regulatory functions in macrophage polarization, inflammatory responses, and tumor microenvironment remodeling (20). Although several reviews have postulated that Kla may be involved in the regulation of bone mineral density, the specific target proteins and signaling pathways remain largely unexplored. Aldo-keto reductase family 1 member A1 (*AKR1A1*) is an important enzyme involved in aldehyde metabolism and the maintenance of redox homeostasis (21). Zhou et al. reported that *AKR1A1* regulates oxidative stress and protein S-nitrosylation under diabetic and hyperlipidemic conditions, suggesting a cytoprotective role in metabolic homeostasis (22). Emerging evidence has indicated that *AKR1A1* expression modulates the metabolic activity and differentiation capacity of osteoprogenitor cells; however, its functional role in lactylation has not been examined in the context of bone metabolism (23). To date, no studies have systematically evaluated the lactylation status of *AKR1A1* or its potential role in the metabolic-immune axis of osteoporosis pathogenesis. In this study, we comprehensively focused on the lactylation of *AKR1A1*, integrating multi-omics analyses, single-cell RNA sequencing, and *in vitro* stimulation assays using RAW264.7 macrophages treated with lactate and osteoporotic serum. Our findings elucidate the involvement of *AKR1A1* in the metabolic-immune regulatory axis of osteoporosis, providing both theoretical insights and experimental evidence for its potential as a therapeutic target.

In this study, a total of 1,347 lactylation-related genes were cross-analyzed by integrating five GEO microarray datasets, leading to the identification of 37 differentially expressed lactylation-related genes (DEGs). Subsequently, a predictive model was constructed using 113 combinations of machine learning algorithms, from which 18 genes with high discriminative power were selected. Among them, *AKR1A1* and *RRP1B* demonstrated exceptional classification performance, with AUC values reaching 1.000 in the training set and 0.944 and 0.720, respectively, in two independent validation datasets, indicating stable predictive capabilities. *AKR1A1* was significantly upregulated in osteoporotic samples and exhibited promising diagnostic and subclassification potential. Single-cell transcriptomic analysis further revealed that *AKR1A1* was highly expressed in monocytes and macrophages, suggesting its potential role in modulating the local immune microenvironment within the bone marrow. Protein–protein interaction (PPI) network analysis based on the GeneMANIA database demonstrated that *AKR1A1* was centrally positioned within a highly connected network, functionally associated with glycolysis/gluconeogenesis, pantothenate and CoA biosynthesis, and the p53 signaling pathway. Both Gene Ontology (GO) and Kyoto Encyclopedia of Genes and Genomes (KEGG) enrichment analyses consistently supported its involvement in metabolic regulation. Previous studies have shown that *AKR1A1* maintains redox homeostasis by catalyzing the reduction of reactive aldehyde and ketone intermediates and plays a cytoprotective role under conditions of oxidative stress and metabolic dysregulation (24). Under hyperglycemic conditions, *AKR1A1* modulates macrophage metabolism and inflammatory status, implicating its dual role in metabolic-immune crosstalk (25, 26). In parallel, lactate-induced protein lactylation has been demonstrated to regulate macrophage polarization, inflammatory transcriptional programs, and bone homeostasis (27, 28). In conjunction with our findings, these results suggest that the lactylation of *AKR1A1* may mediate its immunometabolic regulatory function, thereby playing a critical role in the pathogenesis of osteoporosis.

In our *in vitro* experiments using RAW264.7 cells, both lactate (LAC) stimulation and serum derived from osteoporotic patients (OP) independently induced a significant increase in *AKR1A1* lactylation levels. Notably, the combined treatment with OP serum and lactate (OP+LAC) exerted a synergistic enhancing effect. RT-qPCR and Western blot analyses demonstrated that the mRNA and protein expression levels of *AKR1A1* were highest in the OP+LAC group, significantly exceeding those observed in the single-treatment groups (P < 0.05), indicating that lactate and pathological stimuli cooperatively upregulate *AKR1A1* expression. Furthermore, Co-immunoprecipitation (Co-IP) assays measuring the pan-Kla/*AKR1A1* ratio revealed a significant increase in lactylation in the OP+LAC group (P < 0.01), providing the first experimental evidence of lactate-dependent post-translational modification of *AKR1A1*. These results support the notion that lactate functions not merely as a metabolic byproduct, but as a functional epigenetic modulator involved in protein modification and signaling regulation. Previous studies have demonstrated that lactate-induced protein lactylation in macrophages can activate specific inflammatory gene programs and modulate transcriptional activity via p300-mediated mechanisms (29, 30). Our findings, for the first time, identify *AKR1A1* as a direct target of lactylation, with its modification levels driven by the synergistic effects of lactate and pathological signals, highlighting a distinct physiological–pathological interplay. This conclusion addresses a critical knowledge gap regarding the lactylation of *AKR1A1* and its regulatory role in the context of osteoporosis.

Single-cell RNA sequencing analysis revealed a highly specific expression pattern of *AKR1A1* within bone marrow–derived immune cells, predominantly localized in monocytes and macrophages, suggesting its potential role in the regulation of the osteoporotic immune microenvironment. Integrating these findings with CIBERSORT-based immune infiltration analysis, we observed a significant increase in M1 macrophage proportions in osteoporotic samples, indicating a shift toward pro-inflammatory polarization. The immune cell correlation heatmap further demonstrated a marked negative correlation between T cell subsets (e.g., CD4+ naive and memory resting) and M1/M2 macrophages, implying that T cell functionality may be dynamically influenced by macrophage activity within the bone marrow. Previous studies have confirmed that M1 macrophages, in the context of osteoporosis and inflammatory bone loss, can facilitate osteoclastogenesis and bone resorption by secreting pro-inflammatory cytokines such as *IL-1β* and *TNF-α* (31). Notably, a recent single-cell level study identified an OLR1+ macrophage subset (Mac_OLR1) in osteoporotic bone marrow tissues exhibiting typical M1 characteristics, accompanied by the activation of chemokine and osteoclast-associated signaling pathways, thereby contributing to osteoclast recruitment and microenvironmental remodeling (1). Moreover, lactate metabolism has been shown to modulate macrophage polarization profiles and induce the expression of AKR family enzymes, positioning these enzymes as potential metabolic–immune regulatory hubs (28, 32). Our study further links *AKR1A1* lactylation to specific immune phenotypes, suggesting that it may mediate inflammatory amplification during M1 polarization via a metabolic enzyme–epigenetic modification–signaling axis.

Cell–cell communication analysis based on CellChat revealed a markedly enhanced interaction frequency between macrophages and monocytes exhibiting high *AKR1A1* expression. Ligand–receptor pair identification highlighted the *SPP1–CD44* axis as the dominant signaling pathway. Specifically, *SPP1* was highly expressed in macrophages, whereas its receptor *CD44* was markedly upregulated in monocytes, suggesting a core interactive bridge between these two immune subsets. Supporting this finding, bubble plots, chord diagrams, and pathway heatmaps consistently demonstrated a prominent communication density along this bidirectional axis. Contribution analysis further confirmed that the *SPP1–CD44* signaling route held the highest weight in the overall communication network. In this context, macrophages acted primarily as signal “senders” and monocytes as predominant “receivers,” reinforcing the directionality and functional polarity of this intercellular communication axis. Previous studies have established that *SPP1* plays a critical role in osteoclast adhesion and differentiation, promoting bone resorption via activation of the osteoclastogenesis pathway (33). *CD44*, a widely expressed adhesion molecule on stem and immune cells, has been implicated in macrophage migration and intercellular adhesion processes (34, 35). Moreover, the *SPP1–CD44* axis has been shown to drive macrophage polarization and immune signal transduction in both tumor and tissue immune microenvironments, influencing immune cell infiltration and local inflammatory states (36). This study, for the first time, links *AKR1A1* lactylation—a candidate immunometabolic marker—to the *SPP1–CD44*-mediated bone marrow immune communication network, proposing the “*AKR1A1*–*SPP1–CD44*” axis as a novel signaling pathway involved in osteoporosis pathogenesis.

Multi-omics enrichment analyses revealed that high *AKR1A1* expression was significantly associated with multiple metabolic pathways, including drug metabolism, bile acid biosynthesis, fructose metabolism, and phenylalanine metabolism, encompassing glucose, lipid, and amino acid metabolism. Concurrently, KEGG pathway enrichment also implicated immune-related mechanisms such as the *p53* signaling pathway and T cell receptor signaling, suggesting that *AKR1A1* may exert a coupling regulatory function at the interface between energy metabolism and immune responses. Both GSEA and GSVA analyses consistently supported the potential regulatory role of *AKR1A1* in the metabolic dysregulation underlying osteoporosis. Previous studies have demonstrated that bone metabolism is intricately dependent on mitochondrial activity, glycolysis, and nutrient stress responses, with metabolic reprogramming emerging as a core pathological component of osteoporosis (5, 37, 38). In both osteoblasts and osteoclasts, remodeling of metabolic pathways directly influences their differentiation and functional states (39). *AKR1A1*, as a member of the aldo-keto reductase family, has been established to play a critical role in maintaining redox homeostasis and detoxification of metabolic intermediates (21, 40). However, its mechanistic involvement within the metabolic network of bone tissue remains largely unexplored. This study is the first to propose that *AKR1A1*-mediated lactylation is closely linked to metabolic reprogramming, thereby constructing a tripartite regulatory framework of “lactylation–metabolism–immunity” and offering a novel perspective on the metabolic pathology of osteoporosis.

This study systematically elucidates the multifaceted role of the lactylation-associated enzyme *AKR1A1* in the pathogenesis of osteoporosis and, for the first time, proposes that it orchestrates disease progression through metabolic reprogramming, immune modulation, and intercellular communication. By integrating multi-omics analysis, machine learning-based feature selection, single-cell annotation, and *in vitro* functional validation, a comprehensive research framework was established spanning from biomarker discovery to mechanistic verification. *AKR1A1* lactylation was markedly upregulated in osteoporotic conditions, exhibiting strong diagnostic performance and potential value for molecular subtyping and therapeutic intervention. These findings broaden the research scope and target landscape of metabolic bone disorders.

Despite the robust findings, several limitations remain in this study. First, the *in vitro* experimental system cannot fully recapitulate the complex cell–microenvironment interactions present in osteoporotic bone tissue, and the physiological relevance of the results requires further *in vivo* validation. Second, the specific lysine residues subjected to *AKR1A1* lactylation have not yet been identified, and the structural basis and functional consequences of these modifications remain unclear. Third, the lack of *AKR1A1* knockdown or mutational intervention experiments limits the causal interpretation of its role in the pathophysiological process. Future studies should aim to construct lactylation-deficient *AKR1A1* mutants to delineate critical modification sites and assess their functional impact. *In vivo* validation using osteoporotic animal models would help confirm the pathogenic role of *AKR1A1* in bone metabolism. Moreover, elucidating its regulatory effects on the dynamic balance between osteoclasts and osteoblasts may offer mechanistic insights. On this basis, the development of selective small-molecule inhibitors targeting *AKR1A1* lactylation could represent a novel therapeutic strategy for osteoporosis.

## 5 Conclusion

This study identifies *AKR1A1* as a key lactylation-modified gene involved in the pathogenesis of osteoporosis. Through integrated multi-omics analysis, machine learning-based feature selection, single-cell transcriptomic annotation, and *in vitro* functional validation, we demonstrate that *AKR1A1* is significantly upregulated and exhibits enhanced lactylation under osteoporotic conditions. It is predominantly expressed in monocytes and macrophages, where it participates in metabolic reprogramming, immune polarization, and *SPP1–CD44*-mediated intercellular communication. Functional enrichment analyses further reveal strong associations between *AKR1A1* and glycolytic as well as inflammatory signaling pathways. Collectively, these findings suggest that *AKR1A1* lactylation plays a central role in the metabolism–immunity regulatory axis of osteoporosis, and highlight its potential as an early molecular biomarker and therapeutic target.

## Data Availability

The original contributions presented in the study are included in the article/supplementary material. Further inquiries can be directed to the corresponding authors.
